# Perilesional edema diameter associated with brain metastases as a predictive factor of response to radiotherapy in non-small cell lung cancer

**DOI:** 10.3389/fonc.2023.1251620

**Published:** 2023-10-17

**Authors:** Oscar Arrieta, Laura Margarita Bolaño-Guerra, Enrique Caballé-Pérez, Luis Lara-Mejía, Jenny G. Turcott, Salvador Gutiérrez, Francisco Lozano-Ruiz, Luis Cabrera-Miranda, Andrés Mauricio Arroyave-Ramírez, Federico Maldonado-Magos, Luis Corrales, Claudio Martín, Ana Pamela Gómez-García, Bernardo Cacho-Díaz, Andrés F. Cardona

**Affiliations:** ^1^ Thoracic Oncology Unit, Department of Thoracic Oncology, Instituto Nacional de Cancerología (INCan), México City, Mexico; ^2^ Radioncology Department, Hospital Medica Sur, México City, Mexico; ^3^ Medical Oncology Department, Hospital Medica Sur, México City, Mexico; ^4^ Radiotherapy Unit, Instituto Nacional de Cancerología (INCan), México City, Mexico; ^5^ Oncology Department, Hospital San Juan de Dios, San José, Costa Rica; ^6^ Thoracic Oncology Unit, Alexander Fleming Institute, Buenos Aires, Argentina; ^7^ Neuro-oncology Unit, Instituto Nacional de Cancerología (INCan), México City, Mexico; ^8^ Direction of Research and Education, Luis Carlos Sarmiento Angulo Cancer Treatment and Research Center - Cancer Treatment and Research Cente (CTIC), Bogotá, Colombia

**Keywords:** central nervous system, tumor diameter, perilesional edema, lung adenocarcinoma, lung cancer, local therapy, radiation therapy

## Abstract

**Background:**

Different prognostic scales exist in patients with brain metastasis, particularly in lung cancer. The Graded Prognostic Assessment for lung cancer using molecular markers (Lung-molGPA index) for brain metastases is a powerful prognostic tool that effectively identifies patients at different risks. However, these scales do not include perilesional edema diameter (PED) associated with brain metastasis. Current evidence suggests that PED might compromise the delivery and efficacy of radiotherapy to treat BM. This study explored the association between radiotherapy efficacy, PED extent, and gross tumor diameter (GTD).

**Aim:**

The aim of this study was to evaluate the intracranial response (iORR), intracranial progression-free survival (iPFS), and overall survival (OS) according to the extent of PED and GT.

**Methods:**

Out of 114 patients with BM at baseline or throughout the disease, 65 were eligible for the response assessment. The GTD and PED sum were measured at BM diagnosis and after radiotherapy treatment. According to a receiver operating characteristic (ROC) curve analysis, cutoff values were set at 27 mm and 17 mm for PED and GT, respectively.

**Results:**

Minor PED was independently associated with a better iORR [78.8% vs. 50%, OR 3.71 (95% CI 1.26–10.99); *p* = 0.018] to brain radiotherapy. Median iPFS was significantly shorter in patients with major PED [6.9 vs. 11.8 months, HR 2.9 (95% CI 1.7–4.4); *p* < 0.001] independently of other prognostic variables like the Lung-molGPA and GTD. A major PED also negatively impacted the median OS [18.4 vs. 7.9 months, HR 2.1 (95% CI 1.4–3.3); *p* = 0.001].

**Conclusion:**

Higher PED was associated with an increased risk of intracranial progression and a lesser probability of responding to brain radiotherapy in patients with metastatic lung cancer. We encourage prospective studies to confirm our findings.

## 1 Introduction

Lung cancer (LC) is the leading cause of cancer-related mortality worldwide, with 1.8 million deaths in 2020 and an estimated incidence of 2.2 million (1). Non-small cell lung cancer (NSCLC) represents 85% of LC cases and is the leading cause of brain metastases (BM) (2). Patients with oncogenic driver alterations own the highest cumulative incidences of BM, with a lifetime prevalence between 46% and 80% (3, 4). Other factors associated with a higher BM incidence are the adenocarcinoma subtype, solid predominant tumors (5), disease burden, and carcinoembryonic antigen (CEA) levels (6).

In patients with LC, BM is associated with significant morbidity, mortality, reduced quality of life, and a substantial economic burden (7–9). Some Latin American regions have barriers to LC attention, increasing the mortality of patients with BM (10). Whole-brain radiation therapy (WBRT) has been the standard approach for local control in LC patients, and it remains the preferred modality in case of multiple lesions or symptomatic disease, with a significant improvement in symptom relief, local and distant recurrences, and response rates of 70%–93% (11–13). Nevertheless, WBRT is associated with detrimental effects on cognitive function, no overall survival (OS) benefit, and a median OS of 3–6 months (14).

Stereotactic radiosurgery (SRS) has emerged as a radiation modality for a limited number and size of BM, with high rates of local control comparable with WBRT, lower incidence of neurocognitive effects, and increased overall quality of life (11). Despite the broad introduction of target therapy and immunotherapy in actionable and non-actionable driver gene NSCLC, WBRT remains the most common upfront radiotherapy modality with extremely heterogeneous clinical outcomes (14–16). However, considering the current high effectiveness of CNS-penetrant systemic treatments and novel radiotherapy techniques, selecting patients suitable for local therapy has become controversial.

Extensive efforts have focused on predicting survival outcomes for NSCLC with BM. In this context, several prognostic indexes, such as Recursive Partitioning Analysis (RPA), Graded Prognostic Assessment (GPA) (17, 18), and recently an update of the Disease-Specific GPA using molecular markers (Lung-molGPA index), which introduced EGFR, ALK, and PD-L1 status (19, 20), have been developed to estimate survival and guide clinical decision-making. Nevertheless, limited advances in clinical predictors of radiation therapy response in patients with BM-NSCLC have been studied (11).

The PED has been linked to hypoxia, and HIF1α production promotes pro-angiogenic pathways and the induction of neovascularization, which limits response to radiation therapy (21). In this regard, perilesional edema diameter (PED) has been associated with worse radiological responses and increased risk of new brain lesions in patients with NSCLC (22, 23). A clinical surrogate marker of a radioresistant phenotype might represent a potential predictive factor associated with treatment failure that could help to design highly effective strategies at a central nervous system (CNS) level, minimizing toxicity and extending survival. This study aimed to evaluate the impact of PED on the intracranial response (iORR) and their association with survival outcomes in NSCLC patients with BM receiving radiation therapy.

## 2 Materials and methods

### 2.1 Patient selection

An institutional research committee reviewed and approved the study under project 2021/054.

A retrospective cohort study was conducted between 2014 and 2021. The clinical characteristics, histopathological diagnostic, molecular status, and systemic treatment details were examined through shared medical records. Eligible patients were those with recurrent or metastatic histologically proven NSCLC and measurable intracranial disease according to the Response Assessment in Neuro-oncology (RANO) working group criteria (24), and those who underwent radiation therapy after BM diagnosis. The RANO criteria define measurable disease as bidimensional contrast-enhancing lesions with defined margins, with two perpendicular diameters of at least 10 mm, visible on ≥2 axial slices. Patients with previous local BM-directed treatment (including surgical resection or former radiation treatment), meningeal carcinomatosis, and lack of baseline MRI were omitted.

### 2.2 MRI acquisition and measurements

**Figure 1:**
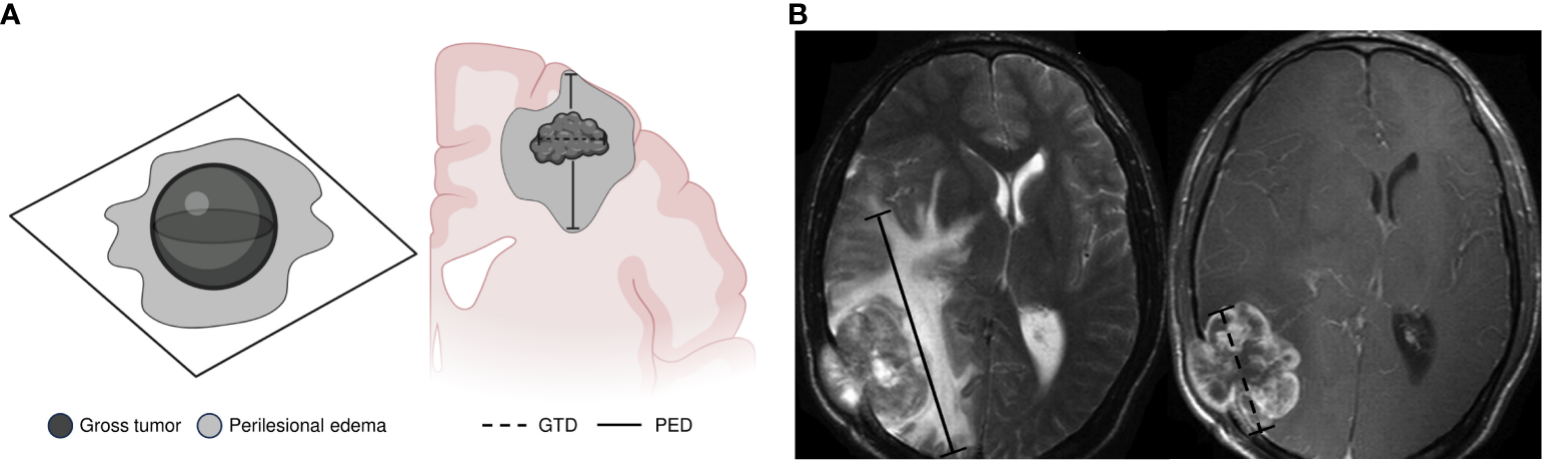
Method used for measuring peritumoral edema extent. We selected the most representative images based on the tumor’s maximum diameter and maximum edema extent on midplane axial, sagittal, or coronal sections. **(A)** Gross tumor (GT) was established as the sum of the maximum diameter (mm) of the three most representative T1-weighted gadolinium-enhanced lesions. Perilesional edema (PE) was defined as the sum of the maximum diameter (mm) at the perilesional hyperintense area on a T2-weighted or FLAIR MRI sequence. **(B)** Examples of perilesional edema diameter (PED) measurement on MRI FLAIR sequences (axial) and gross tumor diameter (GTD) measurement on MRI T1 gadolinium-enhanced scans (axial). GT was established as the sum of the maximum diameter of the three most representative lesions. PED was defined as the sum of the maximum diameter of the perilesional hyperintense area. Dotted black lines indicate the maximum GTD in millimeters (mm). Solid black lines indicate the maximum PED in mm. The PED/GT ratio was calculated by dividing the PED maximum extent by the maximum tumor diameter. GT, gross tumor; GTD, gross tumor diameter; PED, perilesional edema diameter.

All MRI scans were performed on a 1.5-T Signa HDxt scanner (GE Healthcare). Routine MRI pulse sequences included axial T1-, T2-weighted, and fluid-attenuated inversion recovery (FLAIR). A radiation oncologist evaluated baseline neuroimaging features before local therapy. The most representative images were selected based on tumor maximum diameter and maximum edema extent on midplane axial, sagittal, or coronal sections. GT was established as the sum of the maximum diameter (mm) of the three most representative T1-weighted gadolinium-enhanced lesions. In contrast, PED was defined as the sum of the maximum diameter (mm) of the perilesional hyperintense area on a T2-weighted or FLAIR MRI sequence. The PED/GT ratio was calculated by dividing the PED maximum extent by the maximum tumor diameter. **Figure 1** provides guidance on how clinicians performed the measuring method in this study, which could help to incorporate PED in further treatment decisions.

### 2.3 Radiation treatment

Treatment decisions were made on a case-by-case basis by a multidisciplinary tumor board. SRS was performed on the Gamma Knife Radiosurgery platform (Elekta, Stockholm, Sweden). Treatment plans were generated from thin-slice MRI merged with a stereotactic computed tomography scan. SBRT was delivered in cases with up to four intra-axial metastatic lesions, and the regimen was a single dose of 18–24 Gy, resulting in an equivalent dose (EQD2) of 42–68 Gy. The prescription could vary on tumor size and location according to the RTOG-90-05 protocol (25).

Whole brain radiation therapy (WBRT) was delivered as a palliative therapy with a conventional megavoltage external beam radiotherapy administered with a linear accelerator (energy 6 MV). According to the institutional protocol, for all patients treated with WBRT, the regimen was 30 Gy in 10 fractions, resulting in an EQD2 of 32.5 Gy and a biologically effective dose (BED) of 39 Gy, estimated with an alpha/beta ratio of 10. An expert radio-oncologist prescribed hippocampal avoidance, corticosteroid dose, and duration courses.

### 2.4 Outcome measures

The main outcome was the intracranial objective response rate (iORR), defined as the proportion of patients who achieved complete response (CR) and partial response (PR) according to the RANO criteria. Secondary outcomes included the intracranial clinical benefit rate (iCBR) determined as the sum of CR, PR, and stable disease (SD); intracranial duration of response (iDoR) defined as the time from radiation therapy to intracranial progression or death; and depth of response (iDpR) defined as the percentage of maximal tumor reduction from the baseline of intracranial target lesions. Patients were grouped into four quartiles based on the most significant proportion of reduction in intracranial target lesions from the baseline. They were compared with patients with no tumor reduction (NTR). A brain contrast-enhanced MRI was performed at baseline and 8 to 12 weeks after radiation therapy, then every 4 to 6 months or as clinically indicated. An independent radiation oncologist reviewed all response assessments. Intracranial progression-free survival (iPFS) was defined as the time from radiation therapy until intracranial progression or death. OS was defined as the time from radiation treatment until death from any cause.

### 2.5 Statistical analysis

Continuous variables were summarized as arithmetic means with standard deviations or medians with their respective interquartile range. Categorical variables were reported as numbers and percentages. Comparisons between two independent groups were made using the Student’s *t*-test or nonparametric Mann–Whitney *U*-test, according to data distribution determined by the Kolmogorov–Smirnov test. The chi-square test (*χ* ^2^) and the Fisher exact test assessed the differences between the categorical variables as a function of the size of the groups within the comparisons. The quantitative variables were defined as pre-established dichotomous variables and were modeled using bivariate and multivariate logistic regression. The performance of PED and GT diameters to discriminate iORR was assessed by receiver operating characteristic (ROC) curve analyses. Cutoff values were optimal when the product of sensitivity and specificity was maximal. Cutoff point values discriminate between major and minor lesions (mm) related to the edema and tumor. The results were presented as odds ratios (ORs) with corresponding 95% confidence intervals (CIs) and *p*-values and were modeled using bivariate and multivariate logistic regression. Progression-free survival and OS results were analyzed using the Kaplan–Meier estimate, whereas the log-rank test was used to estimate differences among subgroups. All variables were dichotomized for the survival analysis. Predefined variables were chosen for the adjusted multivariate Cox regression model. Hazard ratios (HRs) and their corresponding 95% CIs were calculated to measure association. Statistical significance was set at a *p*-value ≤ 0.05, two-tailed. All statistical analyses were conducted using Stata/MP 14.0 for Mac (Stata Corp LP, 2015), and GraphPad Prism 9.0.1 for macOS (GraphPad Software, 2021) was used for plotting.

## 3 Results

A total of 114 patients with NSCLC and BM diagnosis were identified and eligible for the analysis (**Figure S1**). Patients’ baseline clinical and pathological characteristics and the Lung-molGPA index are summarized in **Table 1**. The mean age was 57.5 ± 12.4 years, and 62 (54.4%) were men. At BM diagnosis, 70 (61.4%) patients had a Karnofsky Performance Status (KPS) of ≤80. The most common histological subtype was adenocarcinoma [98 (86.0%)]; 33 (28.9%) patients harbored a sensitive EGFR mutation (del exon 19 or L858R) or an ALK rearrangement. Only 51 (44.7%) patients had a Lung-molGPA score ranging from 1.5 to 2. Additionally, 83 (72.8%) had multiple lesions in brain MRI, with a median of 2 (1–5) brain lesions. Among these, 43 (37.7%) had five or more BM at diagnosis, and 79 (69.3%) were in the supratentorial compartment. Steroids were employed in 48 (42.1%) of the 114 cases at baseline MRI evaluation. At the MRI response assessment, 28 (43.1%) of the 65 patients used steroids. WBRT was used in 103 (90.4%) patients and SRS in 11 (9.6%). The median prednisone dose did not significantly affect the entire court or between the two groups.

**Table 1:** Demographic and histological characteristics of patients.

		Overall	Perilesional edema diameter (PED), mm		*p*-value
			Minor PED <27	Major PED ≥27	
**Patients**		*n* = 114	*n* = 51	*n* = 63	
**Age, years**	<65	78 (68.4)	36 (70.6)	42 (66.7)	
	≥65	36 (31.6)	15 (29.4)	21 (33.3)	
		57.5 ± 12.4	56.8 ± 12.3	58.1 ± 12.5	0.581
**Sex**	Female	52 (45.6)	32 (62.7)	20 (31.7)	
	Male	62 (54.4)	19 (37.3)	43 (68.3)	**0.001**
**KPS at BM diagnosis**	90–100	44 (38.6)	31 (60.8)	13 (20.6)	
	≤80	70 (61.4)	20 (39.2)	50 (79.4)	**<0.001**
**BM occurrence**	Synchronous	77 (67.5)	31 (60.8)	46 (73.0)	
	Metachronous	37 (32.5)	20 (39.2)	17 (27.0)	0.165
**Smoking status**	Present	58 (50.9)	20 (39.2)	38 (60.3)	
	Absent	56 (49.1)	31 (60.8)	25 (39.7)	0.025
**Histology**	Adenocarcinoma	98 (86.0)	48 (94.1)	50 (79.4)	
	Squamous cell carcinoma	7 (6.1)	1 (2.0)	6 (9.5)	
	Other	9 (7.9)	2 (4.0)	7 (8.3)	0.236
**Adenocarcinoma classification**	Lepidic	9 (7.9)	5 (10.4)	4 (8.0)	
	Acinar	34 (29.8)	18 (37.5)	16 (32.0)	
	Papillary	13 (11.4)	7 (14.6)	6 (12.0)	
	Micropapillary	4 (3.5)	1 (2.1)	3 (6.0)	
	Solid	35 (30.7)	16 (33.3)	19 (38.0)	
	NOS	3 (2.6)	1 (2.1)	2 (4.0)	0.869
**Mutation status**	Wild type/Unknown	81 (71.1)	27 (52.9)	54 (85.7)	
	EGFR/ALK-positive	33 (28.9)	24 (47.1)	9 (14.3)	**<0.001**
**Lung-molGPA index**	0.0–1.0	33 (28.9)	5 (9.8)	28 (44.4)	
	1.5–2.0	51 (44.7)	26 (51.0)	25 (39.7)	
	2.5–3.0	26 (22.8)	16 (31.4)	10 (15.9)	
	3.5–4.0	4 (7.8)			**<0.001**
**Number of BM**	1	31 (27.2)	20 (39.2)	11 (17.5)	
	2–4	40 (35.1)	17 (33.3)	23 (36.5)	
	≥5	43 (37.7)	14 (27.5)	29 (46.0)	**0.023**
**BM location**	Supratentorial	79 (69.3)	41 (80.4)	38 (60.3)	
	Infratentorial	35 (30.7)	10 (19.6)	25 (39.7)	**0.021**
**Steroids at baseline MRI evaluation**	Present	48 (42.1)	12 (23.5)	36 (57.1)	
	Absent	66 (57.9)	39 (76.5)	27 (42.9)	**<0.001**
**Steroids at MRI response assessment (*n* = 65)**	Present	28 (43.1)	11 (33.3)	17 (53.1)	
	Absent	37 (59.9)	22 (66.7)	15 (46.)	**0.087**
**Median dose of prednisone at MRI response assessment (*n* = 65)**		15.0 [5.0–25.0]	20.0 [5.0–45.0]	5.0 [5.0–20.0]	0.578*
**RT modality**	WBRT	103 (90.4)	42 (40.8)	61 (59.2)	
	SRS	11 (9.6)	9 (17.6)	2 (3.2)	**0.012**‡
**Median gross tumor diameter (GTD), mm**		28.1 [15.7–49.1]	17.0 [9.1–26.2]	40.6 [27.5–60.6]	<0.001*

Data are reported as numbers and percentages, n (%), otherwise as mean ± standard deviation or median with interquartile range [IQR]. Normal distribution was tested by Kolmogorov–Smirnoff. Normal distribution assuming not equal variances was analyzed using independent-samples Student´s t-test; otherwise, *Mann–Whitney U-test was applied. Nominal variables were analyzed by the Pearson Chi-Square test, except where a small size (n < 5) was required using ‡ Fisher’s exact test. Two-tailed significance was set at p ≤ 0.05 (bold values). ALK, anaplastic lymphoma kinase; BM, brain metastases; EGFR, epidermal growth factor receptor; KRAS, Kirsten rat sarcoma viral oncogene homolog; KPS, Karnofsky performance status; Lung-molGPA, Graded Prognostic Assessment for NSCLC using molecular markers; RT, radiotherapy; WBRT, whole-brain radiotherapy; SRS, stereotactic radiosurgery. SD, standard deviation. IASLC/ATS/ERS Lung Adenocarcinoma classification (n = 98).

### 3.1 Discriminating values of gross tumor diameter and perilesional edema diameter as potential predictive factors

The best discriminating GTD cutoff value was 17 mm, with a sensitivity of 26.1% and a specificity of 76.2% [AUC = 0.658 (0.44–0.73)] (**Figure S2A**). The best discriminating PED cutoff value for iORR was 27 mm, with a sensitivity of 34.8% and a specificity of 73.8% [AUC = 0.60 (0.46–0.75)] (**Figure S2B**).

### 3.2 Major perilesional edema diameter and clinical characteristics

A major PED (≥27 mm) was identified in 63 (55.2%) patients and was significantly associated with male patients, KPS ≤ 80, multiple brain lesions located in the supratentorial compartment, and more use of steroids at diagnosis. From patients with major PED, 61 (59.2%) were treated mainly with WBRT, and 11 (44.4%) had a Lung-MolGPA score of 0–1. Patients with a PED ≥27 mm had a median GTD larger than those with PED < 27 mm (40.6 vs. 17.0 mm) (**Table 1**).

### 3.3 Intracranial objective response rate

**Figure 2:**
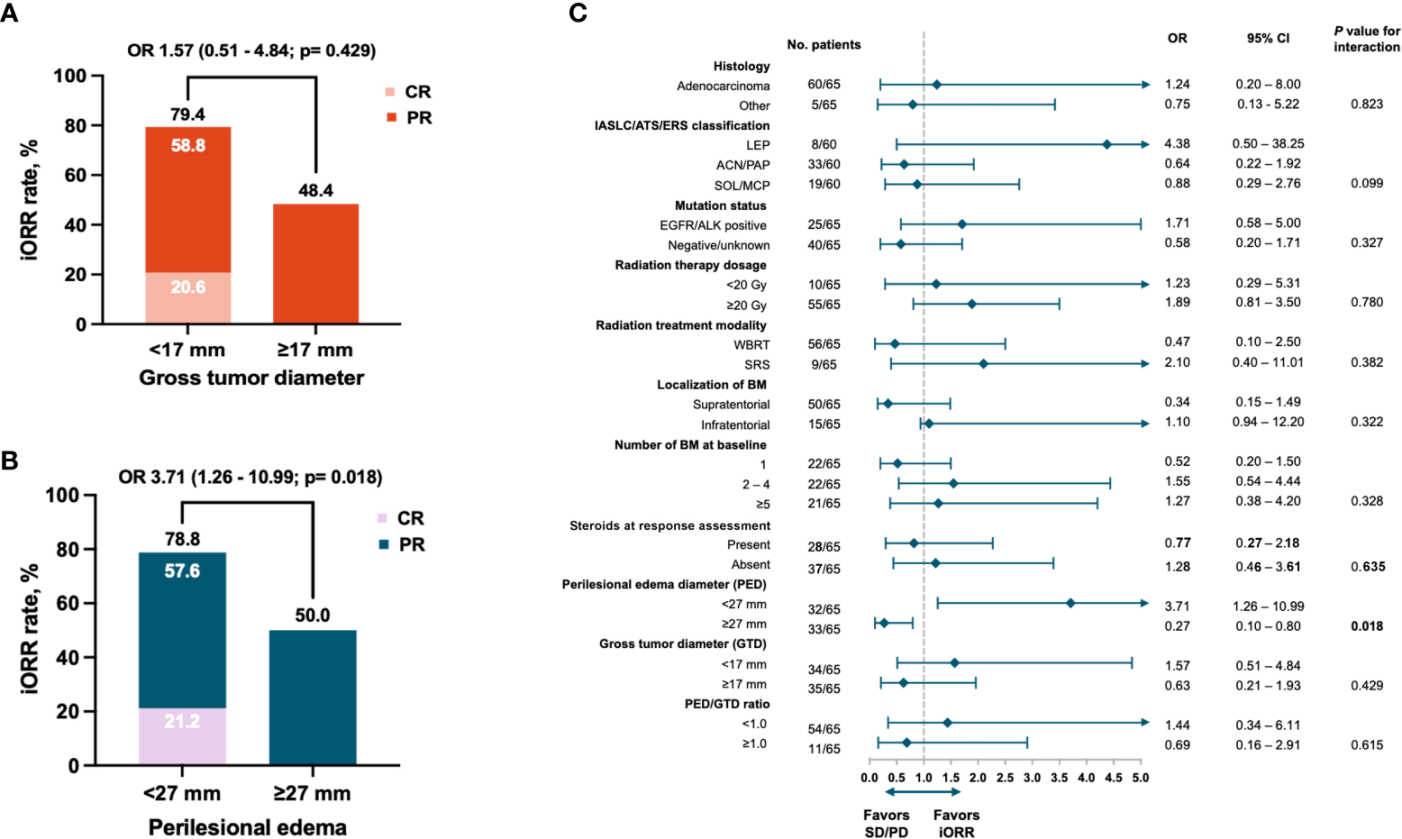
Intracranial response rate according to **(A)** gross tumor diameter, **(B)** Perilesional edema diameter, and **(C)** Forest plot of odds ratios random effects for ICR. Black diamonds and horizontal lines correspond to the ORs and 95% confidence intervals. The two-way solid arrow at the bottom of the graph represents the combined odds ratio and 95% confidence interval. The dotted vertical line corresponds to no effect of response to treatment (odds ratio 1.0). Two-tailed P values ≤ 0.05 were considered statistically significant (Bold values). CR, complete response; PR, partial response; SD, stable disease; PD, progressive disease; PED, perilesional edema diameter; GTD, gross tumor diameter.

The iORR was assessed by a post-RT MRI within 8–12 weeks, and post-radiation therapy was available in 65 (57.0%) cases of the entire cohort. The investigator-assessed iORR was 64.6%. The iORR was significantly higher among patients with minor PED [78.8% vs. 50.0%, OR 3.71 (1.26–10.99; *p* = 0.018)]; however, the GTD showed no significant association (**Figures 2A, B**). In the bivariate analysis, only a minor PED was associated with better intracranial responses [OR 3.71, (95% CI 1.26–10.99); *p* = 0.018) (**Figure 2C**).

**Figure 3:**
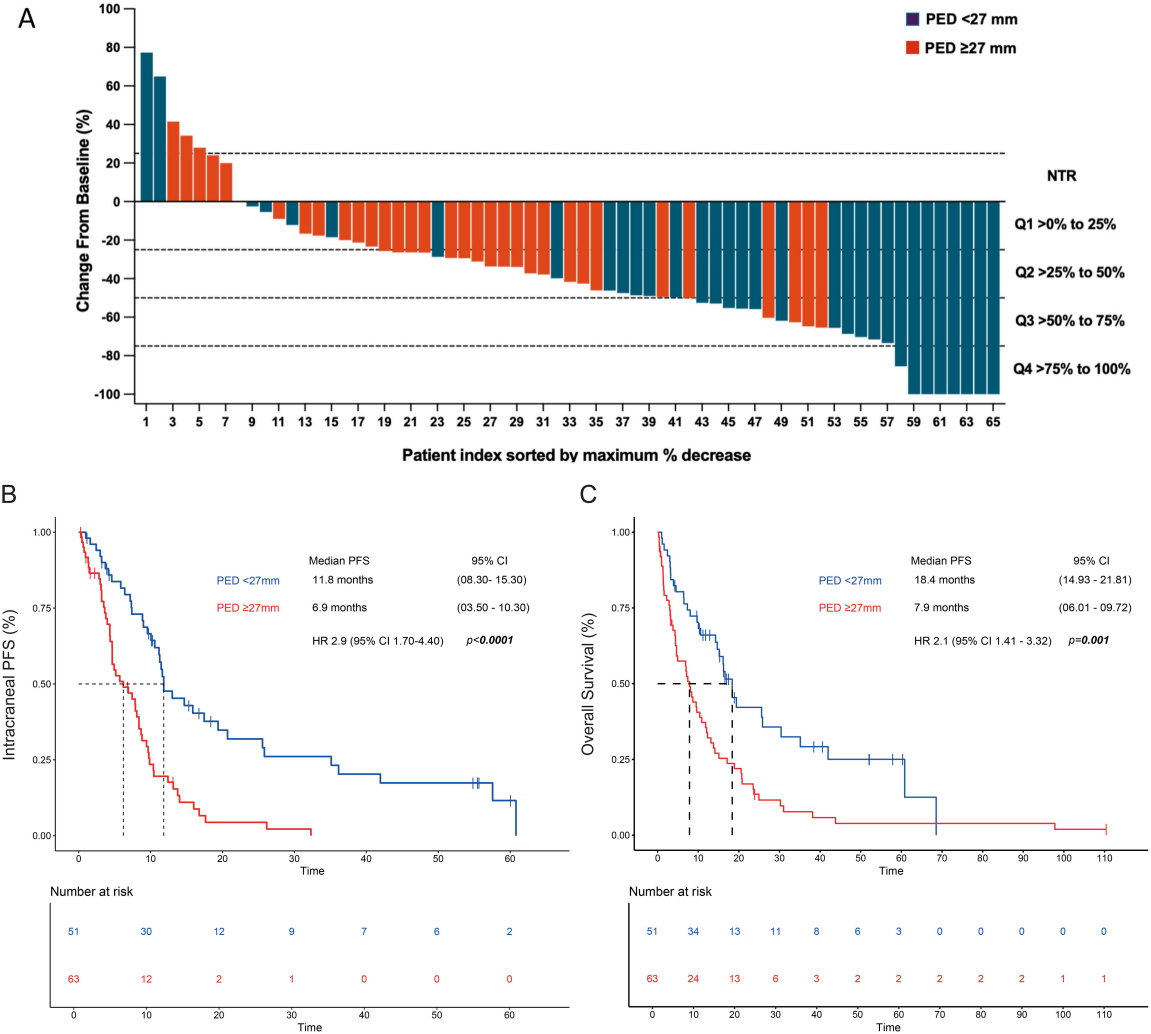
Intracranial responses according to the perilesional edema diameter. **(A)** CNS depth of intracranial responses according to the PED diameter. The Kaplan-Meier plot assessed the PED after radiotherapy according to **(B)** progression-free survival and **(C)** overall survival. MV analysis: PED (>27 mm) remained significant for 8 CNS PFS after the adjustment for sex, Lung-molGPA score, and gross tumor diameter. Cut Off PED was set at <27mm and ≥27mm. Two-tailed P values ≤ 0.05 were considered statistically significant (Bold values). PED, perilesional edema diameter; GTD, gross tumor diameter.

Partial response was reached in 35 (53.8%) patients and showed no significant difference between PED subgroups. Patients who responded utterly belonged to the minor PED subgroup (21.2%, *p* < 0.006). The median iDoR was 9.5 (4.5–14.5) months, with 36 (55.4%) patients achieving a durable response ≥ 6 months. When PED subgroups were compared, the median iDoR was longer in those with a minor PED [13.3 (5.9–20.7) vs. 4.5 (1.2–7.9); *p* < 0.001]. The proportion of patients with a durable response ≥ 6 months favored the subgroup with a minor PED [24 (72.7%) vs. 12 (37.5%), *p* = 0.004]. In patients with any reduction of tumor diameter, DpR was more pronounced among cases with a minor PED. A tumor reduction ≥ 75% was only observed in the subgroup with a minor PED [8(24.2%); *p* = 0.006] (**Table 2**). The maximum percentage change in target lesion size after radiotherapy according to the PED size at baseline is summarized in **Figure 3A**.

**Table 2:** Intracranial response in evaluable population.

	Overall	Perilesional edema diameter (PED)		*p*-Value
		<27 mm	≥27 mm	
Patients	*N* = 65	*N* = 33	*N* = 32	
Investigator-assessed response				
Intracranial objective response (iORR)	42 (64.6)	26 (78.8)	16 (50.0)	**0.015**
Complete response (CR)	7 (10.7)	7 (21.2)		**0.006**
Partial response (PR)	35 (53.8)	19 (57.6)	16 (50.0)	0.540
Stable disease (SD)	15 (23.0)	5 (15.2)	10 (31.3)	0.124
Progressive disease (PD)	8 (12.3)	2 (6.1)	6 (18.8)	0.120
Intracranial disease control rate (iDCR)	57 (87.7)	31 (93.9)	26 (81.3)	0.120
iDoR median (95% IC), mo	9.5 [4.5–14.5]	13.3 [5.9–20.7]	4.5 [1.2–7.9]	**<0.001**
Durable response (CR + PR) ≥6 mo	36 (55.4)	24 (72.7)	12 (37.5)	**0.004**
Depth of response (DpR) category				
NTR	8 (12.3)	3 (9.1)	5 (15.6)	0.423
Q1 > 0% to 25%	10 (15.4)	3(9.1)	7 (21.9)	0.153
Q2 > 25% to 50%	23 (35.4)	8 (24.2)	15 (46.9)	0.056
Q3 > 50% to 75%	16 (24.6)	11 (33.3)	5 (15.6)	0.098
Q4 > 75% to 100%	8 (12.3)	8 (24.2)		**0.003**

Data are reported as numbers and percentages, n (%). Two-tailed significance was set at p ≤ 0.05 (bold values). CNS, central nervous system; iORR, intracranial objective response rate; CBR, clinical benefit rate; BOR, best overall response rate; iDoR, duration of intracranial response; DpR, depth of response; NTR, no tumor reduction; mo, month.

### 3.4 Intracranial progression-free survival

At the data cutoff (22 July 2022), the median follow-up time was 10.2 [3.2–18.9] months. The median iPFS was 8.9 months [7.3–10.5], and the 6-month iPFS rate was 57.0% for the entire cohort (*n* = 114). According to the extension of PED, median PFS was 11.8 (8.3–15.3) months in the group with a minor PED versus 6.9 (3.5-10.2) months in those with a major PED [HR 2.9 (1.7–4.4); *p* < 0.001] (**Figure 3B**).

The 6-month icPFS rate was also higher in the minor PED subgroup, 81.6% (95% CI 67.6 -89.9) versus 50.8% (95% CI 36.9 -63.1, *p* < 0.001, respectively. On bivariate analysis, factors associated with a higher hazard for intracranial progression or death were male patients, a Lung-molGPA index of 0–1 and 1.5–2.0, infratentorial lesions, major PED, and no tumor response to radiotherapy (**Table 3**).

**Table 3:** Bivariate and multivariate analysis (model 1) of progression-free and overall survival.

	Progression-free survival						Overall survival						
	Bivariate analysis			Multivariate analysis			Bivariate analysis			Multivariate analysis			
	HR	95 CI%	*p*	HR	95 CI%	*p*	HR	95 CI%	*p*	HR	95 CI%	*p*	
Sex													
Female	0.47	0.30–0.72		0.646	0.37-1.10	0.111	0.55	0.36–0.85		1.0		
Male	2.15	1.37–3.36	**0.001**	1.0			1.81	1.18–2.77	**0.006**	1.21	0.70–2.08	0.486
IASLC/ATS/ERS adenocarcinoma subtype													
LEP	0.64	0.29–1.40					0.69	0.30–1.60		1.0		
ACI/PAP	1.01	0.64–1.60					0.78	0.49–1.23		0.869	0.50–1.48	0.607
SOL/MIP	1.20	0.75–1.89	0.265				1.49	0.94–2.38	0.089	1.56	0.59–4.09	0.364
Lung-molGPA index													
0.0–1.0	3.81	2.25–6.45		34.85	3.91-310.6	**0.001**	5.27	3.26–8.54		11.89	2.27–62.2	**0.003**
1.5–2.0	1.56	1.02–2.41		9.93	1.24-79.6	**0.031**	1.32	0.86–2.02		3.61	0.80–16.2	0.95
2.5–3.0	0.39	0.23–0.64		3.16	0.39-25.1	0.277	0.23	0.13–0.42		0.727	0.15–3.4	0.685
3.5–4.0	0.11	0.09–0.77	**<0.001**	1.0			0.29	0.07–1.19	**<0.001**	1.0		
Radiation treatment modality													
WBRT	1.84	0.95–3.58		1.27	0.61-2-61	0.514	1.70	0.88–3.30				
SRS	0.51	0.24–1.06	0.071	1.0			0.32	0.13–0.77	**0.012**			
BM location													
Supratentorial	0.59	0.38–0.93					0.63	0.41–0.96				
Infratentorial	1.69	1.08–2.66	**0.022**				1.60	1.04–2.48	**0.034**			
Perilesional edema diameter (PED), mm													
≥27 <27	2.88 0.35	1.83-4.55 0.22–0.55	**<0.001**	3.13 1.0	1.68-5.84	**<0.001**	2.23 0.47	1.34-3.27 0.31–0.73	**0.001**	1.85 1.0	0.93–3.66	0.76
Gross tumor diameter (GTD), mm													
<17	0.78	0.50–1.23		0.518	0.278-0.968	**0.039**	0.88	0.56–1.39		0.471	0.23–0.95	**0.036**
≥17	1.28	0.82–2.00	0.287	1.00			1.13	0.72–1.78	0.593	1.00		
PED/GTD ratio													
<1.0	0.61	0.15–2.52					0.90	0.29–2.89				
≥1.0	1.61	0.40–2.82	0.503				1.09	0.35–3.49	0.872			
Best overall response													
CR + PR	0.57	0.33–0.99					0.52	0.29–0.92				
SD + PD	1.74	1.00–3.02	0.050				1.93	1.08–3.45	**0.026**			
DpR category													
NTR	3.68	1.67–8.16					3.54	1.60–7.88				
Q1	0.89	0.43–1.88					1.06	0.51–2.22				
Q2	1.28	0.72–2.26					0.81	0.44–1.52				
Q3	0.83	0.46–1.56					0.76	0.37 -1.59				
Q4	0.46	0.20–1.07	**0.007**				0.77	0.32–1.81	**0.034**				

DpR, depth of response; PS, performance status; WBRT, whole brain radiotherapy; SRS, stereotactic radiosurgery; CR, complete response; PR, partial response; SD, stable disease; PD, progressive disease; IASLC, International Association of the Study of Lung Cancer; ATS, American Thoracic Society; ERS, European Respiratory Society. Histological grading of differentiation provides a simple architectural grading system, most applicable to resection specimens, with grade 1 (well differentiated; lepidic [LEP] predominant), grade 2 (moderately differentiated; acinar or papillary [ACI/PAP] predominant), and grade 3 (poorly differentiated; solid or micropapillary [SOL/MIP] predominant). Two-tailed significance was set at p ≤ 0.05 (bold values).

Multivariate analysis showed that only the Lung-mol GPA and the PED were significant factors associated with the risk of intracranial progression. After the adjustment for significant factors in model 1, a major PED remained a negative risk factor for intracranial progression [HR 3.3 (95% CI 1.6–5.8)] independently of Lung-molGPA [Index of 0–1 HR 34.8 (95% CI 3.9–31.0); and 1.5–2.0 HR 9.9 (95% CI 1.24–79.6)]. In contrast, the GTD <17 mm became a protective factor for intracranial progression [HR 0.51 (95% CI 0.27–0.96)] after the adjustment (**Table 3**). A second multivariate model was built for progression and survival with the components of the Lung-molGPA displayed separately in **Table S1**. The major PED continued to be a negative factor for intracranial progression.

### 3.5 Overall survival

The median OS was 11.8 [95% CI 7.9–15.7] months, with a 6-month OS rate of 64.9% for the entire population. According to PED, median OS was 18.4 (95% CI 14.9–21.8) versus 7.9 (95% CI 6.0–9.7) months [HR 2.1, (95% CI 1.4–3.3); *p* = 0.001] in the minor versus major PED subgroup, respectively (**Figure 3C**). The 6-month OS rate was 80.3% (95% CI 66.5 -88.9) vs. 57.4% (95% CI 44.1 -68.7), *p* = 0.007, favoring those patients with a minor PED.

On bivariate analysis, factors associated with a higher hazard for death were male patients, Lung-molGPA index of 0–1, infratentorial brain lesions, a major PED, and stable or progressive disease as a best intracranial response. In contrast, protective factors for death were using SRS as the chosen radiation modality. On the multivariate analysis in model 1, only the Lung-mol GPA score of 0–1 increased the risk of death [HR 11.8 (95% CI 2.2–62.2)]. In contrast, a GTD < 17 mm was associated with a better OS [HR 0.47 (95% CI 23–0.95); *p* = 0.036]. PED ≥ 27 mm only showed a tendency to increase the risk of death [HR 1.85 (95% CI 0.93–3.66); *p* = 0.76] (**Table 3**).

The PED was not a significant factor for death in the multivariate analysis in model 2. However, a GTD <17 mm continued to be a protective factor for death [HR 0.38 (95% CI 0.15–0.93), *p* < 0.035] after the adjustment by the individual components of the Lung-molGPA score (**Table S1**).

## 4 Discussion

This retrospective single-center study highlights the relevance of PED as a potential biomarker for intracranial response to radiation therapy in patients with NSCLC and BM. Our results strongly indicate a negative predictive role of PED on response and risk of progression. Peritumoral vasogenic cerebral edema is a significant cause of morbidity and mortality in patients with CNS tumors, including NSCLC BM. This condition is associated with blood–brain barrier (BBB) disruption, leakage of plasma fluid and proteins, increased interstitial fluid pressure (IFP), poor tissue perfusion, and inefficient delivery of oxygen, which results in a hypoxic tumor microenvironment (26).

Hypoxia-mediated radioresistance in CNS tumors is mediated by multiple mechanisms related to cell survival, accelerated tumor proliferation, and repopulation ability (27). Hypoxia-inducible factors (HIFs) play a critical role in regulating genes enabling cell survival in hypoxic environments, including those involved in glycolysis, angiogenesis, and expression of growth factors that promote tumor regrowth (28). Hypoxic tumor microenvironment also drives genomic instability and downregulates DNA repair, leading to cancer progression and radioresistance (29). Furthermore, tumor hypoxia is strongly associated with acquired stem cell phenotype expression in which cancer stem cells (CSCs) are characterized by a reduced accumulation of radiation-induced DNA damage, an increased capacity of DNA Damage Response (DDR) pathway repair, and the activation of anti-apoptotic signaling (29). In addition, CSCs also have lower levels of reactive oxygen species (ROS) and tend to overexpress ROS scavengers, which limits the extent of ROS-dependent damage induced by ionizing radiation (30). Such hypothesis would be supported by the observations of a significantly decreased proportion of tumor shrinkage among patients with greater PED extent.

In the response analysis dataset, a negative association was found between PED and iORR, where patients with a higher PED have a decreased likelihood of achieving intracranial response, consistent with previously published reports (22, 23, 31). Moreover, the study showed a significant association between iDpR and long-term outcomes, which clarifies the significant role of intracranial tumor reduction for survival. Additional studies may further evaluate the role of iDpR as a surrogate endpoint for treatment efficacy in BM.

Measurement of PED represents a simple and accessible potential tool for predicting intracranial response following radiation therapy. Furthermore, it could be easily integrated into clinical practice to identify high-risk patients suitable for treatment intensification strategies. It could also support the rationale to combine anti-angiogenesis agents with radiation therapy to reduce peritumoral vasogenic edema and improve outcomes.

In preclinical and clinical models, agents that target the VEGF signaling pathway have the potential to normalize tumor vasculature, decrease permeability, alleviate edema, lower IFP, improve tissue oxygen levels, and enhance the efficacy of cytotoxic therapies like radiation, chemotherapy, or immunotherapy (32–34). Vascular normalization facilitates the delivery of exogenous agents (35) and enhances DNA damage and cell death through increased ROS after irradiation (35). Additionally, it activates tumor immune response by promoting the maturation and activity of dendritic cells and infiltration of T cells (36, 37), minimizing steroid use and facilitating immunotherapy. Few studies have explored the efficacy and safety of radiotherapy (WBRT or SRS) combined with anti-VEGF, especially bevacizumab, demonstrating encouraging response rates and acceptable safety profile but without consistent survival benefit (38–40).

In the survival analysis, the crucial prognostic role of PED for survival outcome was confirmed; however, it did not remain significant after the adjustment for other relevant prognostic factors. Noteworthy, the PED remained significantly associated with iPFS when adjusted by the other clinical–pathological variables. Notably, PED remained independently associated with poorer outcomes even in the presence of robust prognostic indexes, such as Lung-molGPA. Thus, it is worthwhile to explore the addition of PED to established graded predictive assessment models to improve the accuracy of prognosis for NSCLC patients with BM.

The limitations of this study are the retrospective nature design and the limited number of patients, which prevent us from applying more advanced statistical methods to diminish potential bias. Also, the heterogeneity of the analyzed population, including many oncogene-addictive tumors and various therapies involving target therapy, makes our results difficult to generalize. However, despite the fact that one-third of our cohort harbored an oncogene addictive alteration, the EGFR-TKIs employed were first and second generation in most cases; thus, we did not expect that this factor would affect intracranial response and progression-free outcomes due to the limited brain penetration of these drugs. Moreover, our population received different radiotherapy modalities, which can introduce significant biases to drive definitive conclusions. Of note, WBRT was the chosen radiation modality in 90%, which limits driving conclusions in those patients treated with SRS. Finally, the PED measurement was a fundamental difference between prior studies that might compromise the reproducibility of results.

## 5 Conclusion

PED is a strong predictor of response to radiation therapy in NCSCLC BM. Identification of PED might help better tailor therapy in this context and identify candidates for intensification strategies to improve intracranial response. PED could be a user-friendly tool to predict the survival of patients. It is worthwhile to explore the addition of PED to established graded prognostic assessment models. Further studies are needed to validate these findings. The potential efficacy of anti-angiogenic agents in high-risk patients needs further examination through phase III randomized controlled trials.

## Data Availability

The data analyzed in this study is subject to the following licenses/restrictions: Institutional Data Protection laws. Requests to access these datasets should be directed to ogarrieta@gmail.com.
