# Correction: Perilesional edema diameter associated with brain metastases as a predictive factor of response to radiotherapy in non-small cell lung cancer

**DOI:** 10.3389/fonc.2025.1726889

**Published:** 2025-12-03

**Authors:** Oscar Arrieta, Laura Margarita Bolaño-Guerra, Enrique Caballé-Pérez, Luis Lara-Mejía, Jenny G. Turcott, Salvador Gutiérrez, Francisco Lozano-Ruiz, Luis Cabrera-Miranda, Andrés Mauricio Arroyave-Ramírez, Federico Maldonado-Magos, Luis Corrales, Claudio Martín, Ana Pamela Gómez-García, Bernardo Cacho-Díaz, Andrés F. Cardona

**Affiliations:** 1 Thoracic Oncology Unit, Department of Thoracic Oncology, Instituto Nacional de Cancerología (INCan), México City, Mexico; 2 Radioncology Department, Hospital Medica Sur, México City, Mexico; 3 Medical Oncology Department, Hospital Medica Sur, México City, Mexico; 4 Radiotherapy Unit, Instituto Nacional de Cancerología (INCan), México City, Mexico; 5 Oncology Department, Hospital San Juan de Dios, San José, Costa Rica; 6 Thoracic Oncology Unit, Alexander Fleming Institute, Buenos Aires, Argentina; 7 Neuro-oncology Unit, Instituto Nacional de Cancerología (INCan), México City, Mexico; 8 Direction of Research and Education, Luis Carlos Sarmiento Angulo Cancer Treatment and Research Center - Cancer Treatment and Research Cente (CTIC), Bogotá, Colombia

**Keywords:** central nervous system, tumor diameter, perilesional edema, lung adenocarcinoma, lung cancer, local therapy, radiation therapy

There was a mistake in **Figure 3B** and **Figure 3C**, as published. Kaplan-Meier curves were plotted without censored events. The corrected **Figure 3B** and **Figure 3C**, appear below.

**Figure 3:**
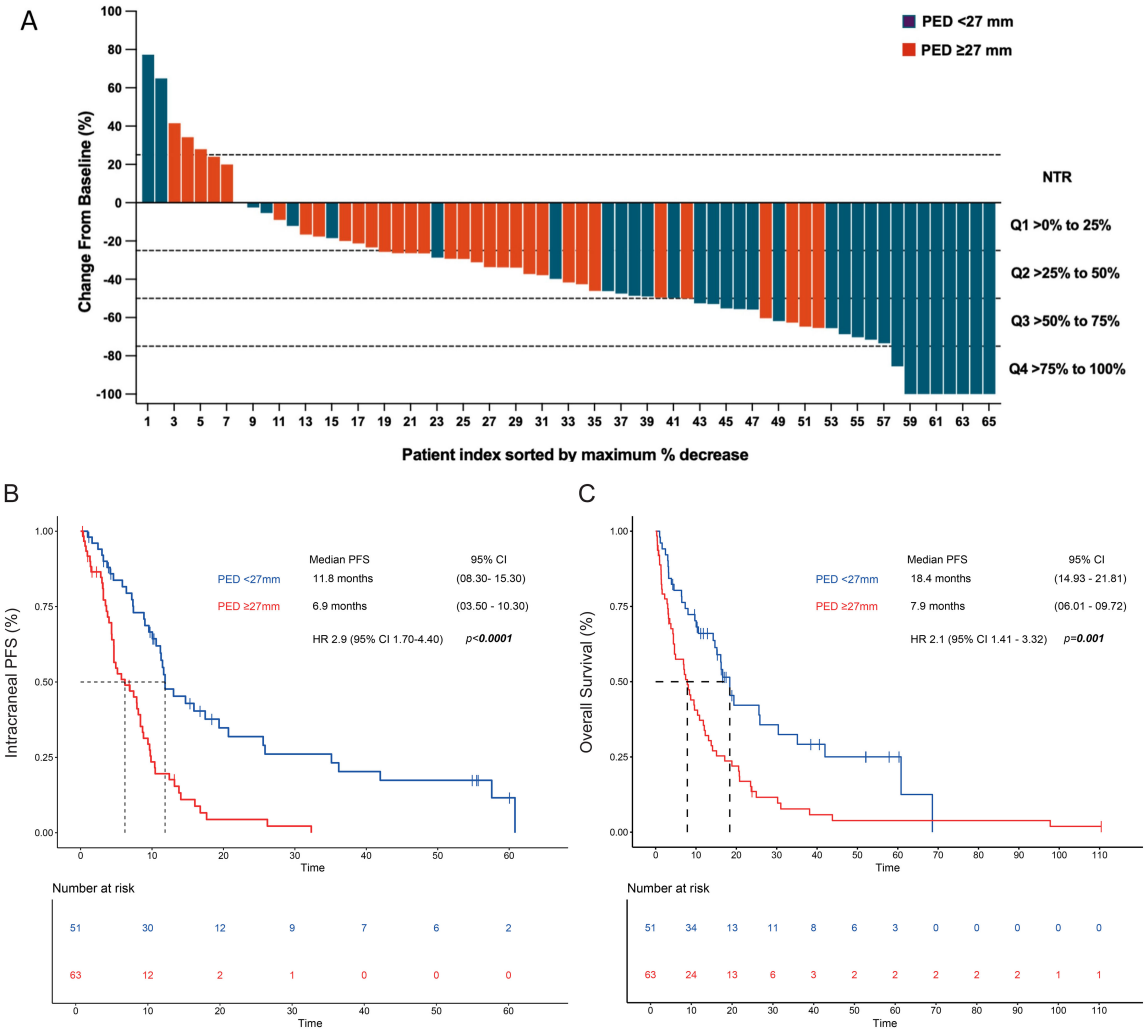
Intracranial responses according to the perilesional edema diameter. **(A)** CNS depth of intracranial responses according to the PED diameter. The Kaplan-Meier plot assessed the PED after radiotherapy according to **(B)** progression-free survival and **(C)** overall survival. MV analysis: PED (>27 mm) remained significant for 8 CNS PFS after the adjustment for sex, Lung-molGPA score, and gross tumor diameter. Cut Off PED was set at <27mm and ≥27mm. Two-tailed P values ≤ 0.05 were considered statistically significant (Bold values). PED, perilesional edema diameter; GTD, gross tumor diameter.

There was a mistake in reported values. A correction has been made to the section *3.4 Intracranial progression-free survival,* third paragraph:

“The 6-month icPFS rate was also higher in the minor PED subgroup, 81.6% (95% CI 67.6 -89.9) versus 50.8% (95% CI 36.9 -63.1, p<0.001, respectively”.

There was a typo mistake in reported values. A correction has been made to the section *3.5 Overall survival,* first paragraph:

“The 6-month OS rate was 80.3% (95% CI 66.5 -88.9) vs. 57.4% (95% CI 44.1 -68.7), p = 0.007, favoring those patients with a minor PED”.

The original version of this article has been updated.

