# Supplementary material for: Perilesional edema diameter associated with brain metastases as a predictive factor of response to radiotherapy in non-small cell lung cancer
Source: Front Oncol. 2023 Oct 17;13:1251620. doi: 10.3389/fonc.2023.1251620 (PMC10616784; doi:10.3389/fonc.2023.1251620)
Supplement: Supplementary file 1 [file Table1.pdf]

Supplementary Table 1. *Bivariate and multivariate analysis (Model 2) of progression-free and overall survival.*

	Progression-free survival						Overall survival					
	Bivariate analysis			Multivariate analysis			Bivariate analysis			Multivariate analysis		
	HR	95 CI%	*P*	HR	95 CI%	*P*	HR	95 CI%	*P*	HR	95 CI%	*P*
**Sex**												
Female	0.47	0.30 – 0.72					0.55	0.36 – 0.85				
Male	2.15	1.37 – 3.36	**0.001**				1.81	1.18 – 2.77	**0.006**			
**Age (years)**												
<70	1.03	0.871 – 1.21					0.75	0.48 – 1.18				
≥70	0.82	0.254 – 2.65	0.740				1.32	0.85 – 2.07	0.215			
**PS at BM diagnosis Karnofsky scale**												
90-100	0.37	0.23 – 0.60		0.449	0.21-0.94	**0.035**	0.32	0.21 – 0.52		0.226	0.103-0.495	**<0.001**
<80	2.68	1.67 – 4.31	**<0.001**	1.00			3.04	1.91 – 4.84	**<0.001**	1.00		
**Histology**												
Adenocarcinoma	0.87	0.45 – 1.69					0.49	0.28 – 0.88				
Other	1.14	0.59 – 2.21	0.691				2.03	1.14 – 3.63	**0.016**			
**IASLC/ATS/ERS adenocarcinoma subtype**												
LEP	0.64	0.29 – 1.40		1.0			0.69	0.30 – 1.60		1.00		
ACI/PAP	1.01	0.64 – 1.60		1.30	0.471-3.63	0.606	0.78	0.49 – 1.23		0.971	0.45-2.05	0.938
SOL/MIP	1.20	0.75 – 1.89	0.265	1.60	0.80-3.19	0.182	1.49	0.94 – 2.38	**0.089**	2.00	0.59-6.78	0.263
**Mutation status**												
EGFR/ALK positive	0.36	0.22 – 0.58		1.0			0.32	0.19 – 0.54		1.00		
Wild type/unknown	2.37	1.47 – 3.83	**<0.001**	3.00	1.50 –6.00	**0.002**	2.79	1.77 – 4.40	**<0.001**	2.63	1.26 – 5.49	**0.010**
**Radiation treatment modality**												
WBRT	1.84	0.95 – 3.58					1.70	0.88 – 3.30				
SRS	0.51	0.24 – 1.06	**0.071**				0.32	0.13 – 0.77	**0.012**			
**Number of BM at baseline**												

1	0.65	0.40 – 1.05		1.00			0.56	0.34 – 0.93		1.0		
2 – 4	1.04	0.69 – 1.60		2.34	1.05-5.21	**0.036**	0.84	0.58 – 1.33		1.50	0.63-3.59	0.355
≥5	1.55	0.97 – 2.50	**0.031**	3.11	1.16-8,38	**0.024**	2.13	1.38 – 3.32	**0.001**	3.75	1.16-12.1	**0.027**
**BM location**												
Supratentorial	0.59	0.38 – 0.93					0.63	0.41 – 0.96		0.66	0.286-1.53	0.338
Infratentorial	1.69	1.08 – 2.66	**0.022**				1.60	1.04 – 2.48	**0.034**	1.0		
**Perilesional edema diameter (PED), mm**												
<27	0.35	0.22 – 0.55		1.00			0.47	0.31 – 0.73				
≥27	2.88	1.83 – 4.55	**<0.001**	2.37	1.06-5.27	**0.034**	2.23	1.34 – 3.27	**0.001**			
**Gross tumor diameter (GTD), mm**												
<17	0.78	0.50 – 1.23		0.484	0.20-1.13	0.093	0.88	0.56 – 1.39		0.38	0.15-0.93	**0.035**
≥17	1.28	0.82 – 2.00	0.287	1.00			1.13	0.72 – 1.78	0.593	1.00		
**PED/GTD ratio**												
<1.0	0.61	0.15 – 2.52					0.90	0.29 – 2.89				
≥1.0	1.61	0.40 – 2.82	0.503				1.09	0.35 – 3.49	0.872			
**Best overall response**												
CR + PR	0.57	0.33 – 0.99		1.0			0.52	0.29 – 0.92		1.0		
SD + PD	1.74	1.00 – 3.02	**0.050**	1.26	0.68-2.33	0.457	1.93	1.08 – 3.45	**0.026**	1.80	0.93-3.46	0.79
**DpR category**												
NTR	3.68	1.67 – 8.16					3.54	1.60 – 7.88				
Q1	0.89	0.43 – 1.88					1.06	0.51 – 2.22				
Q2	1.28	0.72 – 2.26					0.81	0.44 – 1.52				
Q3	0.83	0.46 – 1.56					0.76	0.37 -1.59				
Q4	0.46	0.20 – 1.07	**0.007**				0.77	0.32 – 1.81	**0.034**			

Components of the Lung-molGPA are presented splitted. Abbreviations: EGFR: Epidermal growth factor receptor. DpR: Depth of response. PS: Performance status. WBRT: Whole brain radiotherapy. SRS: Stereotactic radiosurgery. CR Complete response. PR: Partial response. SD Stable disease. PD: Progressive disease. Histological grading of differentiation provides a simple architectural grading system, most applicable to resection specimens, with grade 1 (well differentiated; lepidic [LEP] predominant), grade 2 (moderately differentiated; acinar or papillary [ACI/PAP] predominant), and grade 3 (poorly differentiated; solid or micropapillary [SOL/MIP] predominant). Two-tailed significance was set at $p \leq 0.05$ (Bold values).
