# Supplementary material for: Perilesional edema diameter associated with brain metastases as a predictive factor of response to radiotherapy in non-small cell lung cancer
Source: Front Oncol. 2023 Oct 17;13:1251620. doi: 10.3389/fonc.2023.1251620 (PMC10616784; doi:10.3389/fonc.2023.1251620)
Supplement: Supplementary file 2 [file Image1.pdf]

Supplementary Material

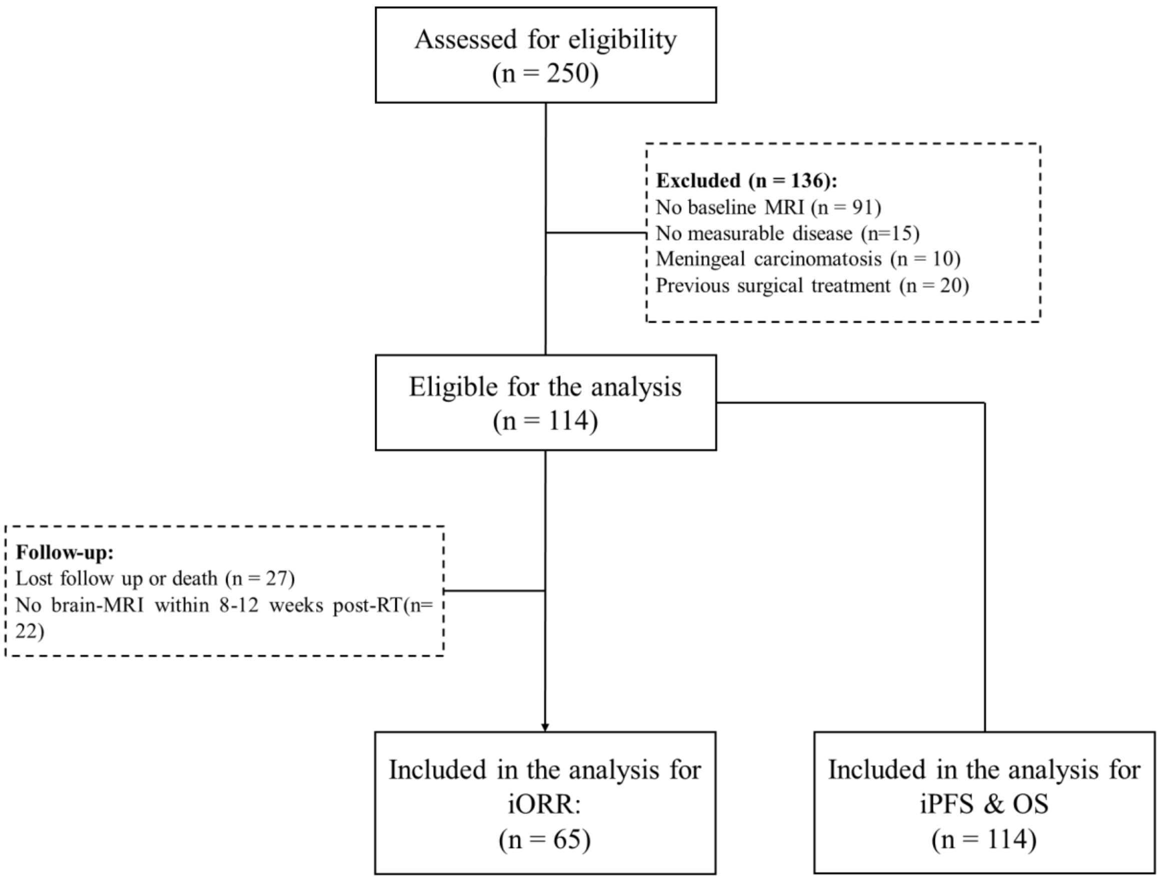

Supplementary Figure 1. STROBE flow chart diagram. Only 65 subjects were evaluable for response with a brain MRI post-RT within 8-12 weeks after radiotherapy. One hundred and fourteen were assessable for iPFS and OS. *MRI: magnetic resonance image; RT: radiotherapy; iPFS: intracranial progression-free survival; iORR: intracranial overall response rate; OS: overall survival.*
