# Supplementary material for: Perilesional edema diameter associated with brain metastases as a predictive factor of response to radiotherapy in non-small cell lung cancer
Source: Front Oncol. 2023 Oct 17;13:1251620. doi: 10.3389/fonc.2023.1251620 (PMC10616784; doi:10.3389/fonc.2023.1251620)
Supplement: Supplementary file 3 [file Image2.pdf]

Supplementary Material

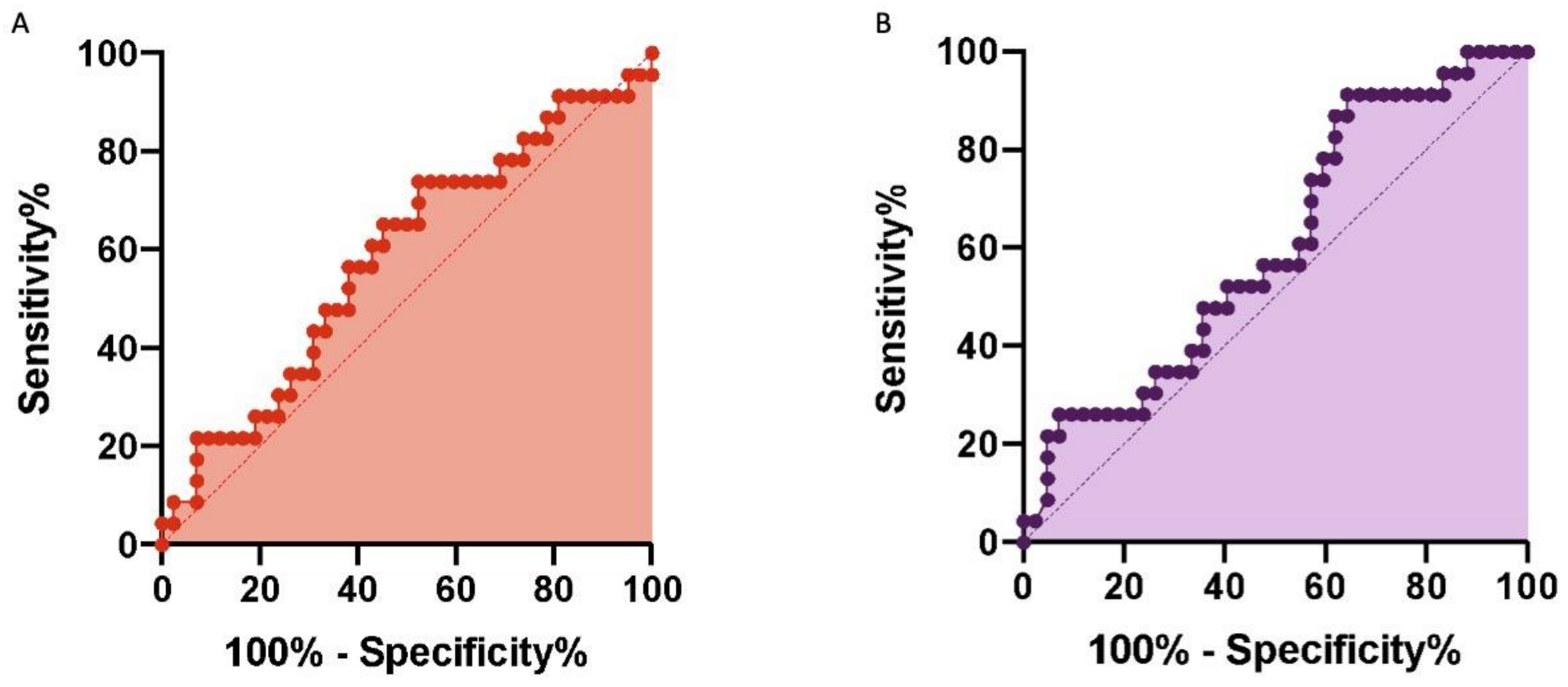

Supplementary Figure 2. Receiver operating characteristic (ROC) curve in brain metastases. **A)** ROC curve demonstrated sensitivity (TRR) and specificity (1-FPR) of the radiotherapy for gross tumor diameter; AUC of 0.58. B) ROC curve demonstrated sensitivity (TRR) and specificity (1-FPR) of the radiotherapy for perilesional edema; AUC of 0.60. Sensitivity = 34.78 and specificity = 73.81. AUC: area under the curve. TOR: true positive rate. FPR: false positive rate.
