# Supplementary material for: Perilesional edema diameter associated with brain metastases as a predictive factor of response to radiotherapy in non-small cell lung cancer
Source: Front Oncol. 2023 Oct 17;13:1251620. doi: 10.3389/fonc.2023.1251620 (PMC10616784; doi:10.3389/fonc.2023.1251620)
Supplement: Supplementary file 4 [file Image3.pdf]

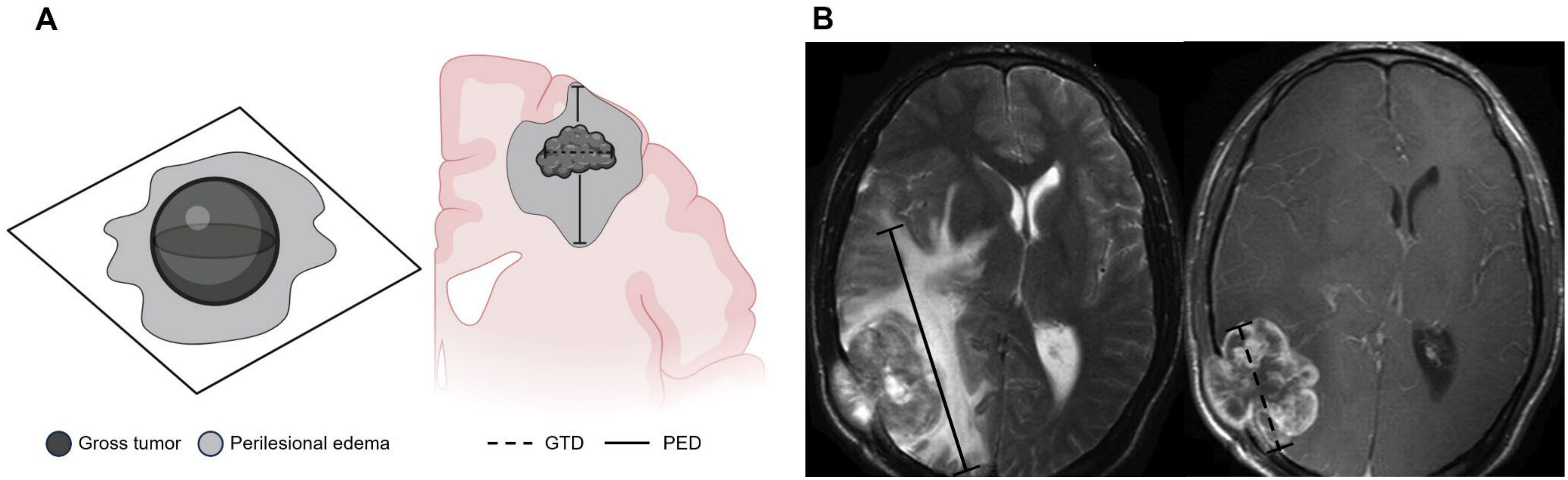

Supplementary Figure 3. Method used for measuring peritumoral edema extent. A) We selected the most representative images based on the tumor's maximum diameter and maximum edema extent on midplane axial, sagittal, or coronal sections. Gross tumor (GT) was established as the sum of the maximum diameter (mm) of the three most representative T1-weighted gadolinium-enhanced lesions. Perilesional edema (PE) was defined as the sum of the maximum diameter (mm) at the perilesional hyperintense area on a T2-weighted or FLAIR MRI sequence. B) Examples of perilesional edema diameter (PED) measurement on MRI FLAIR sequences (axial) and gross tumor diameter (GTD) measurement on MRI T1 gadolinium-enhanced scans (axial). GT was established as the sum of the maximum diameter of the three most representative lesions. PED was defined as the sum of the maximum diameter of the perilesional hyperintense area. Dotted black lines indicate the maximum GTD in millimeters (mm). Solid black lines indicate the maximum PED in mm. The PED/GT ratio was calculated by dividing the PED maximum extent by the maximum tumor diameter. GT: gross tumor; GTD: gross tumor diameter; PED: perilesional edema diameter.
